# Taurine deficiency and dilated cardiomyopathy in golden retrievers fed commercial diets

**DOI:** 10.1371/journal.pone.0209112

**Published:** 2018-12-13

**Authors:** Joanna L. Kaplan, Joshua A. Stern, Andrea J. Fascetti, Jennifer A. Larsen, Hannah Skolnik, Gordon D. Peddle, Richard D. Kienle, Andrew Waxman, Michael Cocchiaro, Catherine T. Gunther-Harrington, Tyler Klose, Kendra LaFauci, Bonnie Lefbom, Maggie Machen Lamy, Rebecca Malakoff, Satoko Nishimura, Maureen Oldach, Steven Rosenthal, Christopher Stauthammer, Lynne O’Sullivan, Lance C. Visser, Regan William, Eric Ontiveros

**Affiliations:** 1 Department of Medicine & Epidemiology, School of Veterinary Medicine University of California Davis, Davis, California, United States of America; 2 Department of Molecular Biosciences, School of Veterinary Medicine University of California Davis, Davis, California, United States of America; 3 Animal Emergency & Referral Associates, Fairfield, New Jersey, United States of America; 4 Mission Valley Veterinary Cardiology, Gilroy, California, United States of America; 5 SAGE Centers for Veterinary Specialty and Emergency Care, Dublin, California, United States of America; 6 Veterinary Specialty Hospital of the Carolinas, Durham, North Carolina, United States of America; 7 Lakeshore Veterinary Specialists, Glendale, Wisconsin, United States of America; 8 CVCA Cardiac Care for Pets Annapolis, Annapolis, Maryland, United States of America; 9 The Regional Veterinary Referral Center, CVCA Cardiac Care for Pets, Springfield, Virginia, United States of America; 10 Veterinary Cardiopulmonary Care Center, West Palm Beach, Florida, United States of America; 11 MSPCA Angell West, Waltham, Massachusetts, United States of America; 12 CVCA Cardiac Care for Pets, Towson, Maryland, United States of America; 13 Department of Veterinary Clinical Sciences, College of Veterinary Medicine University of Minnesota, St. Paul, Minnesota, United States of America; 14 Department of Companion Animals, Atlantic Veterinary College University of Prince Edward Island, Charlottetown, Prince Edward Island, Canada; 15 Veterinary Emergency Clinic, Toronto, Ontario, Canada; University of Illinois, UNITED STATES

## Abstract

**Introduction:**

Golden retrievers are over-represented in cases of taurine-deficient dilated cardiomyopathy and recently a surge in cases has prompted further investigation.

**Objective:**

To describe the clinical, dietary, and echocardiographic features in golden retrievers diagnosed with taurine deficiency and dilated cardiomyopathy, and to determine specific dietary associations. A second aim was to determine the whole blood taurine concentrations in a representative sample of healthy golden retrievers.

**Animals:**

Twenty-four client-owned golden retrievers with documented taurine deficiency and dilated cardiomyopathy and 52 healthy client-owned golden retrievers.

**Methods:**

In this multicenter prospective observational study, baseline and follow-up echocardiographic data, complete diet and medical histories, and whole blood, plasma, or serum taurine concentrations were obtained. Baseline and follow-up echocardiographic data were compared. Associations were evaluated between specific diets and taurine deficiency or congestive heart failure. The prevalence of low whole blood taurine concentrations in the healthy golden retrievers was calculated.

**Results:**

Twenty-three of 24 dogs diagnosed with taurine deficiency and dilated cardiomyopathy were fed diets that were either grain-free, legume-rich, or a combination of these factors. None of these diets were feeding trial tested using Association of American Feed Control Officials (AAFCO) procedures. Twenty-three of 24 dogs had significant improvement in their echocardiographic parameters and normalization of taurine concentrations following diet change and taurine supplementation. Nine of 11 dogs diagnosed with congestive heart failure (CHF) had resolution of their congestion at follow-up with five no longer requiring diuretic therapy and four tolerating diuretic dose reduction by >50%.

**Conclusions:**

Certain diets and diet characteristics were associated with the development of taurine deficiency. Taurine deficiency and dilated cardiomyopathy in golden retrievers is likely multifactorial, including a combination of dietary, metabolic, and genetic factors.

## Introduction

Taurine deficiency and its association with dilated cardiomyopathy (DCM) is well described in the cat and dog [1–10]. In the dog, this sulfur-containing amino acid is synthesized primarily in the liver and central nervous system from methionine and cysteine via the transsulfuration pathway [2]. Due to low enzyme activities of this pathway, which limit endogenous synthesis, taurine is nutritionally essential in the cat, and deficiency was easily and widely accepted as a direct cause of DCM in this species [2–4]. Less intuitive was the discovery that taurine deficiency also leads to DCM in the dog, despite the fact that taurine is not considered an essential amino acid in this species. In 1995, Kramer et al showed that a subset of dogs with DCM, in which golden retrievers and American cocker spaniels were overrepresented, had taurine deficiency [3]. Subsequent studies revealed that when dogs with taurine deficiency and DCM were supplemented with taurine and L-carnitine, several of the myocardial changes resolved, and supportive cardiac medications were removed successfully in the vast majority of these dogs [5–8]. These studies suggested a direct causal link between taurine deficiency and the development of canine DCM. In addition to nutritional etiologies of DCM, several dog breeds have been reported to have an inherited form of DCM. These include breeds like the Doberman Pinscher, Great Dane and Irish Wolfhound [11, 12]. Within these breeds some investigations of association to taurine deficiency have been performed with results that did not clearly indicate any relationship to development of DCM [12].

It has long been recognized that while any breed is at risk of developing DCM secondary to taurine deficiency, certain dog breeds appear to be more susceptible than others, including the golden retriever [3,5–9]. A 2005 case series described taurine deficiency and DCM in five related golden retrievers fed commercial diets consisting of primarily lamb and rice or chicken and rice [5]. In all five dogs, their echocardiographic indices of myocardial function were significantly improved after 3–6 months of taurine supplementation. A limitation of this study was that because these particular golden retrievers were closely related to one other, a hereditary cause for taurine deficiency could not be ruled out and a clear diet association could not be made. In addition to these cases, Fascetti et al. noted three golden retrievers living in the same household with taurine deficiency that were fed a vegetarian diet formulated by the owner [6]. Thus, the preponderance of evidence supports at least some breed-specific sensitivity to taurine deficiency in golden retrievers.

Normal reference ranges for whole blood and plasma taurine can be established based on a small handful of studies published in 2003. In a study by Torres et al, mean +/- standard deviation of plasma and whole blood taurine concentrations in 12 healthy beagle dogs fed purified diets were 109 +/-8 nmol/ml and 291 +/-25 nmol/ml, respectively [13]. A larger study by Delaney et al reported mean +/- standard error of plasma and whole blood taurine concentrations in 131 apparently healthy dogs of a variety of breeds consuming commercial diets to be 77+/-2.1 nmol/ml and 266 +/-5.1 nmol/ml, respectively [14]. Serum taurine concentrations, although less repeatable, in apparently healthy dogs were recently reported to have a median of 178 (range, 110–272) [15]. However, there is little understanding of how plasma and whole blood taurine concentrations correlate with myocardial taurine concentrations in the dog, as endomyocardial biopsy is challenging and not clinically practical. To confound the assessment of taurine concentrations even further, the degree to which breed- or size-specific reference ranges are necessary is unknown but is supported by data that establish specific breeds like golden retrievers, Newfoundlands and American cocker spaniels as having a greater risk for taurine deficiency and subsequent cardiac disease [3,5–9].

Several studies and case reports have established an association between canine taurine deficiency and dietary factors [5–8,16]. In 2001, a study by Sanderson et al was the first to demonstrate that diet could lead directly to taurine deficiency and DCM in the dog [17]. Healthy dogs were fed protein-restricted diets and developed taurine deficiency as a result. One dog that developed both taurine deficiency and DCM had significant improvement in cardiac function with taurine supplementation. Although some protein-restricted veterinary therapeutic diets were supplemented with taurine due to these concerns [9, 16, 17], this practice was not commonplace in over-the-counter dog foods until deficiencies were identified in dogs consuming certain maintenance diets [6,8]. As a result, the prevalence of dilated cardiomyopathy secondary to taurine deficiency was markedly reduced and limited primarily to dogs fed imbalanced, non-commercial, home-cooked or raw food diets. Recently, however, there has been an increase in the number of cases of canine DCM suspected to be related to diet [18].

The purpose of this prospective, observational study was to describe the diet histories, echocardiographic findings, blood taurine concentrations, and clinical outcomes in golden retrievers diagnosed with DCM and low blood taurine concentrations. A second aim of this study is to describe the blood taurine concentrations in a representative population of apparently healthy golden retrievers fed various commercial diets. We hypothesized that specific diets are associated with development of low blood taurine concentrations and taurine-responsive DCM in golden retrievers and that diet change and taurine supplementation would effectively improve the clinical status of these dogs.

## Materials and methods

### Animals and study design

All animals were treated according to standard of care and informed consent for inclusion of patient information in this study was obtained. This is in accordance with the Institutional animal care and use committee of the University of California Davis. Client-owned golden retrievers with documented blood taurine deficiency and dilated cardiomyopathy met inclusion criteria and were enrolled in a multicenter, prospective, observational study to evaluate dietary factors that may contribute to this condition as well as describe their clinical response to treatment. All dogs were recruited from either private or academic institutions in North America from January 2016 to July 2018. All dogs were managed as clinical cases by an attending clinician following standard of clinical care. Client consent for contribution of images and medical records was obtained for all animals in the study. Complete diet histories [19] were obtained for all dogs. The following clinical data were collected for all dogs as part of their clinical management: baseline history, physical examination, standard routine echocardiogram, and either whole blood, plasma, serum, or a combination of whole blood and plasma taurine concentrations. Follow-up data was requested for all dogs if obtained for clinical reevaluation during the study period and included repeat taurine concentrations, updated diet histories, and echocardiograms. All dogs with follow-up data after the initial diagnosis were prescribed a diet change and taurine supplementation at a median dose of 3000mg (range 2000-4500mg) divided two to three times daily as recommended by their attending clinician.

Presence or absence of congestive heart failure was recorded for each dog at each visit. Congestive heart failure was diagnosed when evidence of cardiogenic pulmonary edema was visualized on thoracic radiographs or when pleural or peritoneal effusion were identified on ultrasound with concurrent echocardiographic evidence of dilated cardiomyopathy. Thoracic radiographs were performed at the discretion of the attending clinicians. Treatment recommendations were made at the discretion of the attending clinician, thus medical management was not standardized in cases.

### Echocardiography

All echocardiograms were performed by a board-certified cardiologist or a resident-in-training under the direct supervision of a board-certified cardiologist. All echocardiographic images obtained from outside institutions were sent for off-line analysis by a single investigator (JS) using a commercially available workstation (Syngo Dynamic Workplace, Version 10.0.01_HF04_Rev5 [Build 2884], Siemens Medical Solutions, Malvern Pennsylvania). A diagnosis of DCM was made if echocardiographic measurements and calculations met at least two out of four of the following specific criteria: left ventricular percent fractional shortening (%FS) <25% [20, 21], percent fractional area change (%FAC) <35%, percent ejection fraction (%EF) <40% [22–24] and left ventricular internal diameter at end-systole (LVIDs)> 3.5 cm [20]. Percent fractional shortening and LVIDs were obtained either in M-mode or two-dimensional (2D) imaging from the right parasternal short-axis at the level of the papillary muscles [21]. In addition to the diagnostic criteria for DCM diagnosis, selected echocardiographic indices were measured and recorded for all animals at all visits when available. Left atrial to aortic root ratios (LA:Ao) were obtained from 2D imaging of the right parasternal short axis view at the heart base [25]. Left atrial enlargement was determined by an LA:Ao ratio of ≥1.6. Left ventricular internal dimension at end-diastole (LVIDd) and LVIDs were measured using the leading-edge method at end diastole and systole, respectively. Percent fractional shortening was calculated using the equation (LVIDd-LVIDs)/LVIDd x 100 [21]. An LVIDd >5.1 was criteria for an enlarged left ventricle [20]. Using planimetry, the left ventricular area at end-diastole (LVAd) and end-systole (LVAs) was determined by tracing the endocardial border of the left ventricle in the left apical four-chamber view excluding the papillary muscles. Percent fractional area change was calculated as (LVAd-LVAs)/LVAd x 100. Left ventricular volumes at end-diastole (LVVd) and end-systole (LVVs) were estimated by tracing the endocardial border of the left ventricle in the left-apical four chamber view and employing the modified Simpson’s rule of discs [23, 24]. Percent ejection fraction was calculated as (LVVd-LVVs)/LVVd x 100. Dogs with evidence of concurrent cardiac disease that could lead to a DCM phenocopy (i.e. patent ductus arteriosus, aortic regurgitation or severe myxomatous mitral valve disease) were excluded from the study. Dogs with incomplete imaging that did not permit assessment of presence or absence of DCM criteria were excluded from the study.

### Taurine analysis

Whole blood (lithium heparin), plasma (lithium heparin), or serum (additive-free tube) taurine concentrations were analyzed via commercial laboratories [26–29]. As this was an observational multicenter study, data regarding the volume of blood collected for each sample could not be obtained for all samples. For cases seen at UC Davis, at least 2 ml of blood was obtained for plasma samples and at least 1 ml of blood was obtained for whole blood samples. No serum analyses were performed through the UC Davis Amino Acid Laboratory. All samples were collected prior to initiation of taurine supplementation or diet change. For purposes of this study, in this breed, a low taurine level was defined as a whole blood taurine concentration of ≤ 250 nmol/mL; plasma taurine concentration of ≤ 60 nmol/mL [13, 14] and serum taurine concentration of ≤110 nmol/mL [15]. These concentrations were chosen based upon previously published manuscripts documenting higher mean blood taurine concentrations [13, 14] than currently reported reference ranges [26] as well as the authors clinical observations that golden retrievers with whole blood taurine concentrations between 200-250nmol/mL experienced DCM resolution with diet change and taurine supplementation. Although specific reference ranges for taurine concentrations in golden retrievers has not yet been established, support for breed specific reference ranges has been documented by multiple investigators [3, 5, 7–9, 13, 14]. Dogs with measured taurine concentrations that exceeded the pre-determined cut-off values were excluded from the study.

### Diet history and nutritional analysis

Complete diet histories [19] were obtained for all dogs. Data collected included brand names and varieties of the diet(s) fed at the time of sample collection for baseline taurine concentrations. In addition, daily quantity fed, and length of time (days) the dog had been receiving the baseline diet was recorded. Any medications, supplements, or probiotics the dog was receiving at the baseline visit were noted. When available, if a diet change was made, the same information was recorded. Dogs with incomplete diet histories were also excluded from this study. Each diet manufacturer was coded numerically (1–9) and when manufacturers produced more than one diet identified in the study the separate diets were categorized by an alphabetical letter (a-m).

For each diet, the listed ingredients, caloric content (kcal/cup and kcal/kg), crude fiber as fed (%), crude fiber on a dry matter basis (%), moisture (%), total dietary fiber, insoluble fiber, and soluble fiber content when available were recorded [30, 31]. Actual caloric intake for each dog was recorded and compared to their calculated resting energy requirement (RER) and maintenance energy requirement (MER). The nutritional adequacy statement for each diet was assessed to determine if there was a complete and balanced claim and if so, the method of substantiation for the claim (formulation or feeding trials) per the Association of American Feed Control Officials (AAFCO) [32]. If diets did not undergo a feeding trial, but the pet food label claimed the diet was formulated to meet AAFCO guidelines, it was determined whether or not this was confirmed via formulations or analysis of the finished product based on the World Small Animal Veterinary Association (WSAVA) recommendations [33]. Research included a comprehensive evaluation of the pet food bag, the manufacturer’s website, and phone communications with representatives from both the supplier and manufacturer when available.

The ingredient list for each diet were recorded and assessed. Whether or not the diet was advertised as grain-free was recorded, and diets were considered to have legumes (peas or pea components, lentils, beans, or chickpeas) as a primary ingredient if included in the first five listed ingredients.

The RER was calculated for each dog using the following equation: RER = 70 x BW ^ 0.75, where BW = body weight in kilograms at the time of diagnosis [34]. The MER range for each dog was calculated by multiplying the RER by a factor of 1.4 and 1.6 to account for a more sedentary or more active lifestyle, respectively [35]. Percent differences between amount fed (kcal/d) and calculated MER (kcal/d) were calculated. Percent differences between amount fed (kcal/d) and recommended amount (kcal/d) based on the individual’s weight as instructed by the feeding directions on the diet label or website were also calculated.

### Prevalence of taurine deficiency in golden retrievers

Whole blood taurine concentrations were determined in samples collected from apparently healthy, client owned golden retrievers with no reported history of clinical signs related to cardiac disease. Dog data was obtained as the result of a screening clinic at the Golden Retriever Club of America National Specialty event. All golden retrievers were reported to have no clinical signs of cardiac disease based on client history and sought blood taurine screening for preventive medicine purposes. Complete diet histories were collected for all dogs being screened as described for the clinical cases. In each dog, at least 1 ml of blood was collected from a jugular vein and placed in a lithium heparin tube. Samples were frozen on dry ice and shipped overnight to be stored at -80°C until submitted. All samples were submitted for whole blood taurine analysis the following business day [26].

### Statistical analysis

Statistical analysis was performed with commercially available software (GraphPad Prism 7.0d Macintosh Version by Software MacKiev 1994–2017 GraphPad Software, Inc). All echocardiographic data at baseline and final recorded follow-up visit were tested for normality visually and using the D’Agostino-Pearson omnibus normality test. Column statistics were performed for all variables and reported as mean +/- standard deviation or median (IQR) for parametric or non-parametric data respectively. When applicable, range was reported as minimum (min) and maximum (max) values. Baseline and final follow-up echocardiographic data were compared using either a paired sample t-test (parametric) or Wilcoxon matched-pairs signed-rank test (non-parametric). A P value of less than 0.05 was considered significant.

Correlation analysis using Pearson’s correlation coefficient was performed to determine if severity of taurine deficiency was correlated with selected echocardiographic parameters (% FS, %FAC, %EF, LVIDs, and LA:Ao). All data for this analysis followed a parametric distribution.

Statistical Fisher’s exact tests were computed to determine statistical associations between diet brand and low blood taurine concentrations and between diet brand and study cases of DCM with and without congestive heart failure (CHF). For the former, both clinical cases and healthy dogs were pooled. Taurine concentrations were categorized as normal or low based on cut-off values for whole blood of 250 nmol/ml and for plasma of 60 nmol/ml. These associations were tested for any diets fed to ≥2 dogs with DCM and low blood taurine concentrations within the clinically affected group of 24 dogs.

## Results

A total of 40 dogs were considered for study inclusion. Sixteen dogs were excluded from the study due to one of the following reasons: inadequate imaging to assess DCM status (n = 7), no evidence of dilated cardiomyopathy based on echocardiographic remeasurement by investigator (n = 8), taurine concentrations that were categorized as normal regardless of whether or not they had underlying cardiac disease (n = 1), incomplete diet history (n = 0), or concurrent cardiac disease that was considered hemodynamically significant (n = 0). A total of 24 client-owned golden retrievers diagnosed with taurine deficiency and DCM were enrolled in the clinical portion of this study. Of these dogs, 8/24 were female (5 intact and 3 spayed) and 16/24 were male (8 intact and 8 neutered). The mean age was 6 years +/- 3 years (min 1 year, max 11 years). Mean weight was 32.4 kg +/- 4.7 kg (min 22.9 kg, max 45.3 kg). Body condition scores (BCS) on a 9-point scale [36] were obtained in a total of 20/24 dogs. Mean BCS was 5 +/-1 (min 3 max 7). The majority of dogs (13/20) had an ideal BCS of either 4 or 5.

### Echocardiographic findings

Baseline echocardiographic data obtained in all 24 dogs included left ventricular %FS, LVIDd, LVIDs, and LA:Ao ratio. Baseline %EF and %FAC were obtained in 18/24 dogs. Follow-up echocardiographic data for %FS, LVIDd, LVIDs, and LA:Ao ratio was available in 16/24 dogs, and %EF and %FAC was available in 12/16 dogs. Time elapsed between baseline and follow-up echocardiogram was a median of 250 days (IQR 166–291.5 days, range 26–860 days). Baseline and follow-up echocardiographic measurements are reported as means +/- standard deviations in Table 1.

**Table 1:** Baseline and follow-up mean, standard deviation, and range for echocardiographic parameters.

	Reference Intervals	Baseline Visit			Follow-up Visit			
Echo Indices	Healthy Dogs^18,20–23^	Mean	SD	Range	Mean	SD	Range	P Value
%FS	27–55	16.7	4.5	7.6–22.8	26.0	6.8	13.3–35.1	<0.0001
%FAC	>35	25.9	5.5	14.6–35.1	39.7	9.6	26.4–57.9	<0.0009
%EF	>40	35.3	7.2	18.7–46.8	54.0	11.3	39.5–74.9	<0.0003
LVIDs (cm)	1.8–3.5	5.0	1.0	3.5–7.0	4.1	0.9	3–6.5	<0.0001
LA:Ao	>1.6	1.7	0.4	1.0–2.6	1.4	0.2	1–1.8	<0.0007
LVIDd (cm)	3.7–5.1	6.0	1.0	4.5–7.8	5.5	0.8	4.3–7.5	0.005

%FS = percent fractional shortening, %FAC = percent fractional area change, %EF = percent ejection fraction using the modified Simpson’s rule of discs, LVIDs = left ventricular internal diameter at end-systole, LA:Ao = left atrial to aortic diameter, LVIDd = left ventricular internal diameter at end-diastole. SD = standard deviation. P values < 0.05 are considered significant. Reference intervals established in healthy golden retrievers are available for %FS, LVIDs, and LVIDd^18^. Reference intervals established for %FAC, %EF, and LA:Ao are based on all breeds as breed-specific reference values are not available to the authors’ knowledge [22–25].

All twenty-four dogs met our criteria for DCM at baseline. All dogs had a %FS <25%, 17/18 dogs had a %FAC <35%, 12/18 dogs had a %EF <40%, 24/24 dogs had an LVIDs >3.5 cm, 20/24 dogs had an LVIDd of >5.1 cm, and 12/24 dogs had an LA:Ao ≥1.6. In addition to a diagnosis of DCM, one golden retriever had a restrictive ventricular septal defect while another had concurrent mild subaortic stenosis. However, these were not thought to be clinically or hemodynamically significant and therefore, these dogs were not excluded from the study. All other dogs had no echocardiographic evidence of concurrent chronic valvular disease or congenital cardiac anomalies.

**Fig 1:**
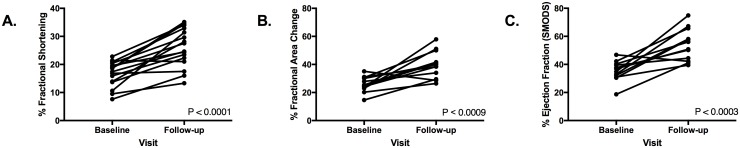
Percent fractional shortening, %FAC, and %EF in each dog from baseline to follow-up visit. P values <0.05 were significant.

**Fig 2:**
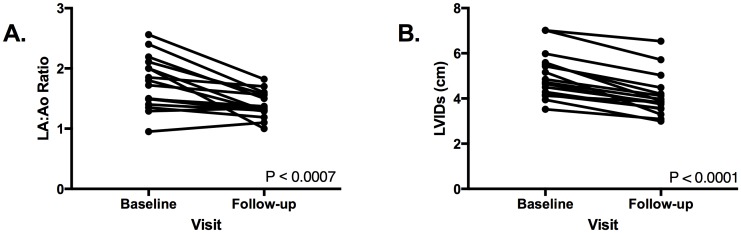
Left atrial-to-aortic root ratio and LVIDs in each dog from baseline to follow-up visit. P values <0.05 were significant.

Percent left ventricular fractional shortening, %FAC, and %EF were significantly increased from baseline to follow-up visits in all but one dog (Fig 1). Furthermore, LA:Ao and LVIDs were significantly decreased from baseline to follow-up visit in all but two dogs and one dog, respectively (Fig 2). Left ventricular internal diameter was increased in 20/24 dogs at baseline. Left ventricular internal diameter in diastole significantly decreased from baseline to follow-up visit in 13/16 dogs.

### Taurine concentrations

Baseline whole blood (n = 16), plasma (n = 4), serum (n = 1), or both whole blood and plasma (n = 3) taurine concentrations were available. Follow-up whole blood (n = 13), plasma (n = 1), or both whole blood and plasma (n = 1) taurine concentrations were provided in 15/24 dogs, collected at a mean of 143 +/-93 days (range: 24–333 days) after diet change and supplementation. In 2/15 of these dogs, the baseline sample was plasma, and the follow-up taurine concentrations were measured in whole blood. In one dog, the baseline sample was serum, and the follow-up taurine concentration was measured in whole blood.

Mean baseline whole blood taurine concentrations of 19/24 dogs was 142.7 +/- 58.19 nmol/ml (min = 48 nmol/ml, max = 240 nmol/ml). Most dogs (16/19; 84%) had whole blood taurine concentrations less than 200 nmol/ml, while 3/19 (16%) dogs had whole blood taurine concentrations between 200 and 250 nmol/ml. Median baseline plasma taurine concentrations in 7 dogs was 28 nmol/mL (IQR 5.3–47). Of interest, the dog with a plasma taurine concentration of 61 nmol/mL had a whole blood taurine concentration of 174 nmol/ml. Therefore, this dog was assessed to have low taurine concentrations, as plasma taurine concentrations are more susceptible to artifactual elevations from improper sample handling.

Follow-up whole blood taurine concentrations were available in 14/24 dogs, with a mean of 413.6 +/- 100 nmol/ml, (min = 213 nmol/ml, max = 604 nmol/ml). Thirteen out of 14 dogs had a follow-up whole blood taurine concentration greater than 250 nmol/ml. The remaining dog out of 14 had a follow-up whole blood taurine concentration of 213 nmol/ml. In addition, a follow-up plasma taurine concentration was available in one of these 14 dogs, measuring 191 nmol/ml. Of interest, the dog with a whole blood taurine concentration measuring 213 nmol/ml is the only dog that demonstrated worsening of systolic function with a reduced left ventricular %FS, %FAC, and %EF from baseline to follow-up visit.

When baseline whole blood taurine concentrations were compared to each baseline echocardiographic parameter individually, the severity of taurine deficiency was not significantly correlated with any single echocardiographic parameter.

### Clinical features and medical therapy for taurine-deficiency and DCM

At baseline, 11/24 dogs were diagnosed with congestive heart failure and prescribed diuretic therapy (furosemide). Nine out of these 11 dogs had resolution of congestion at time of follow-up. Of these nine dogs, five had successful discontinuation of furosemide therapy, and four had a reduction of their maintenance furosemide dose by 50–56%. In the two remaining dogs, one dog remained in congestive heart failure and the other was lost to follow-up. The remaining 13 dogs were considered to have occult DCM and no diuresis was prescribed.

At baseline, 24 dogs were prescribed taurine supplementation at a median dose of 1500mg orally twice per day (daily dose range of 2000-4500mg) and thirteen dogs were additionally prescribed L-carnitine supplementation at a median dose of 2000mg per day (range 500-6000mg). Additional medications included pimobendan (Boehringer Ingelheim Vetmedica, Inc., Duluth, GA, USA) (n = 13), enalapril (n = 7), benazepril (n = 4), spironolactone (n = 6), and diltiazem (n = 2). Follow-up data was available in 16 dogs. One dog was successfully removed from all cardiac medications and remained on only taurine and L-carnitine supplementation. In addition, taurine and L-carnitine supplements were successfully discontinued in four dogs based upon echocardiographic resolution of DCM.

### Non-cardiac medications and supplementation prior to diagnosis

Fifteen out of twenty-four dogs were reported by owners to receive non-cardiac related medications and supplements including various antibiotics (n = 4), mibolerone (n = 1), biotin (n = 2), probiotics (n = 5), multivitamins (n = 4), non-steroidal anti-inflammatories (n = 2), joint supplementation (n = 4), levothyroxine (n = 1), fish oil (n = 3), coconut oil (n = 1), and Oclacitinib (Apoquel; Zoetis Inc., Forham Park, NJ, USA) (n = 1). All reported antibiotics were started either at the time of diagnosis or less than one month prior to diagnosis. No supplement products included taurine or carnitine prior to diagnosis.

### Diet and nutritional analysis

The group of 24 golden retrievers diagnosed with DCM and low taurine concentrations, were fed a total of nine different pet food brands (listed as diets 1–9) and a total of thirteen different varieties (listed as a-m) (Table 2). All diet labels included a complete and balanced claim substantiated by formulation to meet the AAFCO Dog Food Nutrient Profiles; none had undergone feeding trials for nutritional adequacy. No baseline diets met the recommendations of the World Small Animal Veterinary Association (WSAVA) Global Nutrition Committee [33]. Total dietary fiber, insoluble fiber, and soluble fiber concentrations were not available for any diets, confirmed by phone communication with both the pet food suppliers and manufacturers for each diet when available. Percent crude dietary fiber reported by diet company ranged from 2.7% to 9.09%. Of note 15/24 dogs were fed diet 1, 2/24 were fed diet 3, 2/24 were fed diet 5, and 1 dog each was fed each of the remaining diets listed in Table 2. Twelve of 13 diets were grain-free, and 10/13 diets contained legumes within the first five ingredients listed. A Fisher’s exact test revealed a statistically significant (P = 0.0012) association between diet 1 and a low taurine concentration when compared to all other diets (Table 3). The association test between diets 3 and 5 and a low taurine level was not statistically significant (P = 0.51, P = 0.14 respectively). Furthermore, having the clinical sequelae of CHF was not significantly associated with any of the 3 diets fed to at least 2 dogs in the DCM group (diets 1, 3, or 5). The length of time in days that dogs had been fed their diet at the time of diagnosis was available in 22 out of 24 dogs with a median of 814.5 days (min = 182 days, max = 3558 days). In the remaining 2/24 dogs, the length of time was not reported by the client in one dog and the length of time was described qualitatively by the client as “several years” in the other dog.

**Table 2:** List of pet food brands with their diet varieties and characteristics. For each pet food variety, the number of dogs diagnosed with DCM fed this diet and the number of dogs with taurine deficiency fed this diet were listed. G = grain-free diet, L = if a legume is listed as one of the first five ingredients of the diet. Note that one dog on diet 1a is the same dog receiving diet 9m. The one dog receiving diet 8k is the same dog receiving diet 8l.

Diet Brand	Diet Variety	No. of dogs with DCM	No. of dogs with low Taurine	Feeding Trial Tested	Formulated to Meet AAFCO Guidelines	Meets WSAVA Guidelines	G	L
1	a	10	10	No	✓	No	✓	✓
	b	4	4	No	✓	No	✓	✓
	c	1	1	No	✓	No	✓	
2	d	1	1	No	✓	No	✓	✓
3	e	1	1	No	✓	No	✓	✓
	f	1	1	No	✓	No	✓	✓
4	g	1	1	No	✓	No	✓	✓
5	h	2	2	No	✓	No	✓	✓
6	i	1	1	No	✓	No	✓	✓
7	j	1	1	No	✓	No	✓	✓
8	k	1	1	No	✓	No	✓	✓
	l	1	1	No	✓	No	✓	✓
9	m	1	1	No	✓	No	✓	

1a = ACANA Singles Limited Ingredient Diet Pork & Squash Formula (dry); Manufactured

by Champion Petfoods USA Inc., Auburn, KY 42206.

1b = ACANA Singles Limited Ingredient Diet Lamb & Apple Formula (dry); Manufactured by Champion Petfoods USA Inc., Auburn, KY 42206.

1c = ACANA, unknown variety; Manufactured by Champion Petfoods USA Inc., Auburn, KY 42206.

2d = Taste of the Wild Pet Food Pine Forest Canine Recipe with Venison & Legumes (dry); Schell & Kampeter, Inc. Manufactured by Diamond Pet Foods.

3e = 4health Grain Free Beef & Potato Formula (dry); Tractor Supply Co.; Manufactured by Ainsworth Pet Nutrition, Meadville PA 16335.

3f = 4health Grain Free Chicken & Vegetable Formula (dry); Tractor Supply Co.; Manufactured by Diamond Pet Foods.

4g = Zignature Lamb Formula Limited Ingredient (dry); Pets Global, Inc.; Manufactured by Tuffy’s Pet Foods, Perham, MN 56573.

5h = Instinct Limited Ingredient Diet Grain-Free Recipe with Real Lamb (dry); Nature’s Variety, Saint Louis, MO 63146. Manufactured by CJ Foods, Inc, Bern, KS, 66408.

6i = NutriSource Grain Free Chicken and Peas Formula (dry); Manufactured by KLM Family Brands, Tuffy’s Pet Foods, Perham, MN 56573.

7j = Kirkland Signature Nature’s Domain, Turkey Meal & Sweet Potato Formula for Dogs (dry); Manufactured by Diamond Pet Foods.

8k = Fromm Lamb & Lentil Recipe Dog Food (dry); Manufactured by Fromm Family Foods LLC, Mequon WI 53092

8l = Fromm Salmon a La Veg (dry); Manufactured by Fromm Family Foods LLC, Mequon WI 53092

9m = Orijen Regional Red (freeze-dried medallions); Manufactured by Champion Petfoods USA Inc., Auburn, KY 42206.

(✓) Diet meets criteria under the specific category

**Table 3:** Dietary associations between taurine concentrations and congestive heart failure diagnosis.

Diet	Diet vs. Taurine	Diet vs. CHF
**1**	**0.0012**	1.0000
3	0.51	1.0000
5	0.14	0.1993

P values are reported for Fisher’s exact test used to test for associations between the three most common diets fed within the cohort of 24 golden retrievers (diets 1, 3, and 5) and the development of low taurine or congestive heart failure (CHF). A P value of <0.05 is considered significant.

1 = ACANA Manufactured by Champion Petfoods USA Inc., Auburn, KY 42206.

3 = 4health Tractor Supply Company., Brentwood, TN, 37027.

5 = Instinct Limited Ingredient Diet Grain-Free Recipe with Real Lamb (dry); Nature’s Variety, Saint Louis, MO 63146. Manufactured by CJ Foods, Inc, Bern, KS, 66408.

Actual daily caloric intake for baseline diet could be calculated for 23/24 dogs based on the number of cups consumed per day and the caloric content. Only 1 dog was consuming an amount that exceeded the predicted MER; 22/23 of the dogs for which this could be calculated were consuming up to 62% less than the predicted MER using the less active factor applied to RER (Table 4).

**Table 4:** Nutritional requirements and affected dog data.

Subject No.	Weight (kg)	BCS	Diet	Calculated RER (kcal/d)	MER range (kcal/d)	% Difference in actual vs. MER	Bag recommendation (kcal/d)	% Difference in actual vs. bag recommendations
1	27.9	4	1a	971	1360–1554	-9.8 to -21.0	716–1329	-7.7 to 71.4
2	35.34	5	1a	1162	1627–1860	-5.7 to -17.5	818–1534	0 to 87.5
3	35	6	1a	1151	1612–1842	-23.9 to -33.4	818–1534	-20 to 50
4	34.9	5	1a	1149	1608–1838	-11.0 to -22.1	818–1534	-6.66 to 75
5	35.8	5	1a	1171	1639–1873	-0.2 to -12.7	818–1534	6.7 to 100
6	32	6	1a	1076	1507–1722	-32.1 to -40.6	818–1534	-33.33 to 25
7	36.8	6	1a	1195	1673–1912	-38.9 to -46.5	1023–1841	-44.44 to 0
8	33.1	7	1b	1104	1546–1766	-53.7 to -59.5	818–1534	-53.3 to -12.5
9	30	6	2d	1025	1436–1641	-53.1 to -58.9	1264–1517	-46.7 to -55.6
10	29.5	4	1b	1013	1418–1620	-56.7 to -62.1	818–1534	-60 to -25
11	30.2	5	5h	1031	1443–1649	-7.1 to -18.7	1118–1565	-14.3 to 20
12	45.3	*	1a	1397	1956–2235	4.6 to -8.5	1023–1841	11.1 to 100
13	27.7	4	3e	966	1352–1546	-21.7 to -31.5	971	9.1
14	38.8	7	4g	1244	1741–1990	-25.1 to -34.4	1196–1305	0 to 9.1
15	22.9	3	7j	837	1172–1340	-28.4 to -37.3	1008–1344	-37.5 to -16.7
16	34.4	5	8k	1136	1591–1818	-48.7 to -55.1	1224–1469	-44.5 to -33.3
			8l			-50.1 to -56.3	1191–1429	-44.4 to -33.3
17	28.5	5	1b	987	1382–1579	-11.2 to -22.3	716–1329	-7.7 to 71.4
18	27.9	5	1b	971	1360–1554	-9.8 to -21.0	716–1329	-7.7 to 71.4
19	37.6	*	1c	1215	1701–1944	*	*	*
20	29.1	*	1a	1002	1403–1604	-41.7 to -49.0	716–1329	-38.5 to 14.3
21	30.9	5	1a	1048	1468–1678	-8.5 to -19.94	818–1534	-12.4 to 59.8‡
			9m				863	
22	29.75	*	5h	1019	1427–1631	-6.0 to -17.8	1118–1565	-14.3 to 20
23	33	5	3f	1101	1542–1762	-45.0 to -51.9	1695	-50
24	29.6	5	6i	1015	1421–1624	-33.2 to -41.5	1107–1188	-20 to -14.2

List of individual weight, body condition score (BCS), diet brand and variety, calculated resting energy requirement (RER) in kilocalories per day (kcal/d), calculated maintenance energy requirements (MER) in kcal/d, percent (%) difference in actual amount of diet fed (kcal/d) to calculated MER, the recommended amount (kcal/d) to feed based on individual’s weight as instructed by diet bag label or website, and the percent (%) difference in actual amount of diet fed (kcal/d) to the recommended amount (kcal/d) based on individual’s weight as instructed by diet bag label or website. Maintenance energy requirements were listed as a range as RER multiplied by a factor of 1.4 to 1.6 to account for a less active and more active lifestyle, respectively. In several diets, recommended daily calorie intake was listed as a range to account for a less active or more active lifestyle. In a total of five subjects (2, 4, 6, 7, and 23) dogs received a range in the amount of food fed daily. For these dogs, their greatest and smallest amount of diet fed daily were averaged. Subject 16 received diet 8k or diet 8l and therefore, both diets were analyzed separately. Quantity of diet fed to subject 19 was not made available. Subject 21 received equal proportions of diet 1a with diet 9m, and therefore, actual calorie intake was calculated by adding kilocalories contributed from both diets.

^‡^Because subject 21 was fed a combination of diet 1a and diet 9m, daily kilocalories recommended by manufacturer for both diets were averaged to calculate the percent difference between actual amount fed and amount recommended.

*Information not available.

1a = ACANA Singles Limited Ingredient Diet Pork & Squash Formula (dry); Manufactured by Champion Petfoods USA Inc., Auburn, KY 42206.

1b = ACANA Singles Limited Ingredient Diet Lamb & Apple Formula (dry); Manufactured by Champion Petfoods USA Inc., Auburn, KY 42206.

1c = ACANA, unknown variety; Manufactured by Champion Petfoods USA Inc., Auburn, KY 42206.

2d = Taste of the Wild Pet Food Pine Forest Canine Recipe with Venison & Legumes (dry); Schell & Kampeter, Inc. Manufactured by Diamond Pet Foods.

3e = 4health Grain Free Beef & Potato Formula (dry); Tractor Supply Co.; Manufactured by Ainsworth Pet Nutrition, Meadville PA 16335.

3f = 4health Grain Free Chicken & Vegetable Formula (dry); Tractor Supply Co.; Manufactured by Diamond Pet Foods.

4g = Zignature Lamb Formula Limited Ingredient (dry); Pets Global, Inc.; Manufactured by Tuffy’s Pet Foods, Perham, MN 56573.

5h = Instinct Limited Ingredient Diet Grain-Free Recipe with Real Lamb (dry); Nature’s Variety, Saint Louis, MO 63146. Manufactured by CJ Foods, Inc, Bern, KS, 66408.

Twenty-one of 24 dogs were switched to a new diet following a diagnosis of taurine deficiency and DCM. For 3 dogs follow-up data including diet information was not available. No dog was switched to a diet that was reported in the baseline diet histories for the group. Seventeen of 21 switched to a grain-inclusive diet while 4 switched to a different grain-free diet. Only one dog was found to have a persistently low whole blood taurine concentration, despite diet change and supplementation. Of interest, this dog was switched to a unique but still grain-free variety of food with legumes within the top 5 ingredients and with a complete and balanced claim substantiated by the formulation method rather than feeding trials; this diet did not meet the recommendations of the WSAVA Global Nutrition Committee [33].

### Prevalence of low taurine in apparently healthy golden retrievers

Mean whole blood taurine concentration in samples obtained from 52 apparently healthy golden retrievers (mean age 5.1 +/- 2.8; mean weight 27.7 +/- 4.6 kg; 15 females, 13 spayed females, 16 males, and 8 castrated males) was 279.1 +/- 51.5 (min 164 nmol/ml, max 382 nmol/ml). Forty-three of 52 had complete diet histories available. Twelve of 52 dogs had whole blood taurine concentrations of 200–250 nmol/mL, and 4/52 dogs (7.7%) had whole blood taurine concentrations < 200 nmol/mL. Interestingly, all 4 dogs with whole blood taurine concentrations < 200 nmol/mL, and 10 dogs with whole blood taurine concentrations between 200–250 nmol/ml in which complete diet histories were available, were on diets that were legume-rich, and/or were grain-free. None of the diets fed to these 14 dogs met the recommendations of the WSAVA Global Nutrition Committee [33]. Of the 27 dogs with whole blood taurine concentrations > 250 nmol/ml for which complete diet histories were available, 11 (40.7%) were being fed diets that met the WSAVA GNC recommendations, a much higher prevalence than in the group with taurine deficiency (0/14; 0%).

## Discussion

This study represents both the largest scale and longest-term follow-up data available in dogs with low taurine concentrations and concurrent dilated cardiomyopathy. With 24 dogs enrolled and 16 dogs with follow-up data over a one-year period we identify the clinical and echocardiographic features, response to therapy, and dietary similarities among cases.

Significant improvement in echocardiographic parameters and normalization of whole blood taurine concentrations from baseline to follow-up visits were observed in all but one dog after implementing a diet change and supplementation with taurine +/- L-carnitine. This suggests a strong association between low taurine concentrations and the development of dilated cardiomyopathy. This is consistent with previous findings, demonstrating that low plasma taurine concentrations can be associated with DCM in breeds that do not typically develop the hereditary form of the disease [3]. In addition, several publications have documented the echocardiographic resolution of nutritionally-mediated dilated cardiomyopathy as early as one to two months following supplementation with taurine with or without L-carnitine [5–8]. While our study did not specifically aim to identify the time to improvement in echocardiographic parameters, we did note that by a median of 8 months the vast majority of dogs (15 of 16) showed significant echocardiographic improvement.

All but one dog in this cohort of golden retrievers demonstrated significant clinical improvement. In addition, in a total of eleven dogs diagnosed with congestive heart failure, nine had resolution of congestion, and five had successful discontinuation of furosemide. This is consistent with previous reports demonstrating that DCM secondary to taurine deficiency may carry a good prognosis as cardiac changes are potentially reversible [5–8]. One dog in our study remained in congestive heart failure, which may have been due to lack of a diet change, lack of owner compliance in medication or supplement administration, or lack of response to therapy. It should be emphasized that not all cases respond positively. Therefore, prevention of the disease is ideal.

In addition to its major function in the conjugation of bile acids, taurine is thought to play an important role in myocardial health, as it is the most abundant free amino acid in the heart. It has been postulated that taurine is involved in cardiac contractility, modulation of calcium fluxes, and cell membrane stabilization [2,4]. Although DCM is perhaps the best described clinical manifestation, taurine deficiency has also been implicated in retinal lesions and ocular blindness, reproductive failure, growth retardation, central nervous system dysfunction, skeletal spinal malformations, increased platelet aggregation, and neutrophil dysfunction [2]. These additional sequelae were not evaluated in this cohort of dogs.

The causes of taurine deficiency are multifactorial. Although taurine is not considered an essential amino acid in the dog, unlike in humans that can alternatively conjugate bile acids with glycine when taurine is less available, dogs are obligated to conjugate bile acids with taurine [4]. Therefore, it is postulated that this species variation may make dogs more vulnerable to developing taurine deficiency if metabolic demands are high or if taurine is not efficiently reabsorbed by the intestinal tract. Despite the role of taurine in cardiac health, the addition of taurine and dietary testing of taurine concentration is not required of pet food manufacturers.

Taurine status is impacted by several dietary factors [5, 7–8, 13, 17, 37, 38]. For example, it is well established that certain cooking methods can result in taurine losses [38] and that cooking and processing can impact taurine status through effects on the intestinal microbiota [39, 40]. Although all of the baseline diets reported in this study were dry extruded kibble products, processing techniques and conditions applied to the raw materials and to the final product likely varied but are unknown and could not be assessed or compared in this study.

Animal muscle tissue has the highest amounts of taurine compared to other dietary components common to commercial pet foods [38]. The increased use of previously uncommon animal protein sources such as rabbit, venison, bison, lamb, and wild boar, especially in the category of diets marketed as grain-free, warrants characterization of their typical amino acid profiles including the degree and nature of any variability. In fact, certain meats are unexpectedly low in taurine (such as rabbit) [41] or low in sulfur amino acid precursors (such as lamb meal) [42]. In addition, the bioavailability of taurine and its precursors in many animal protein sources is not known. Many of the baseline diets in this study contained animal protein sources that until recently were not commonly found in canine diets. Given the paucity of data available regarding the amino acid composition and bioavailability of many of these ingredients, it is impossible to say with any certainty what role, if any, they may have. Ideally, amino acid concentrations in the diet, especially taurine, methionine, and cystine would be obtained to determine if diets were deficient in any of these essential ingredients. However, this data was not made available by the diet manufacturers. Further research is needed to better understand their characteristics before any additional conclusions can be drawn.

Although plants have little to no taurine [43], in some diets they may provide adequate precursors for taurine synthesis in the dog. Legumes are increasingly included in canine diets as a source of carbohydrate, protein, and fiber, and appeared to be a significant component in the majority of baseline diets reported in this study. However, legumes are limiting in sulfur amino acids, and the anti-nutritional factors they commonly contain such as proteolytic inhibitors and phytates further negatively impact the digestibility and bioavailability of these taurine precursors [44]. Also, legumes are high in fiber, and there is some evidence that certain fiber sources result in depletion of taurine in both dogs and cats [45, 46]. Characterization of the fiber types (concentration of total dietary fiber and proportion of soluble vs insoluble fiber) in the diets consumed at baseline by the dogs in this study was not possible due to lack of information provided by the manufacturers.

Finally, many dogs in this study were consuming not only less than their predicted needs based on calculated MER ranges, but also less than the manufacturer’s feeding directions indicated. This may have been associated with suboptimal intakes of sulfur amino acid precursors in addition to other essential nutrients if diet formulation did not take into account this variability in energy requirement. It has previously been demonstrated that when diets are near-limiting in sulfur amino acids, dogs with lower energy requirements show slower taurine synthetic rates [47] and may therefore benefit from higher dietary concentrations of these taurine precursors. All of the baseline diets in this study had their complete and balanced claim substantiated by formulation to meet the AAFCO Dog Food Nutrient Profiles rather than by feeding trials for nutritional adequacy. Given that diet performance cannot be accurately predicted by use of the formulation method, and at least a subset of the canine population appears to be unable to adequately meet metabolic needs for taurine on some diets, in vivo testing of diets including assessment of taurine status may be indicated and should be encouraged. While this study focuses on dietary similarities between affected dogs and generates statistical associations implicating these diets, the complexities of canine taurine metabolism make causation impossible to prove. Randomized, prospective, controlled feeding trials and other mechanistic research are needed to fully explore the impacts and potential interactions among specific ingredients, nutrient concentrations, fiber sources, processing conditions, and other dietary factors as they relate to taurine status in the dog.

Golden retrievers are overrepresented as a breed at risk of developing taurine deficiency [3,5,6]. Taurine deficiency has been previously associated with certain diets in several breeds, including the golden retriever, Newfoundland, American Cocker Spaniels, English Setter, Labrador retriever, and several others [5–8]. The fact that some breeds have an apparent increased risk for diet-related DCM suggests that there may be genetic or metabolic differences between breeds that make them more vulnerable to developing taurine deficiency. In the sample of fifty-two apparently healthy golden retrievers, there was an unexpectedly high prevalence of low whole blood taurine concentrations, suggesting that the prevalence of taurine deficiency and DCM may be higher in the true population than observed and supporting that golden retrievers may represent a breed-specific sensitivity to this condition. Echocardiograms were not available for these apparently healthy dogs and therefore, it is unknown if any of these dogs had occult DCM.

There are several limitations to this study, a majority of which are related to the enrollment of clinical cases and some variability in clinician-directed treatment protocols. Medical therapy, taurine analysis, baseline and treatment period diets, and supplementation were not standardized across all cases, making it difficult to identify any treatment-specific differences from our results. Currently, although controversial, supplementation with both taurine and L-carnitine is recommended in cases of nutritionally-mediated dilated cardiomyopathy [10]. This is because in previous studies documenting resolution of nutritionally-mediated DCM with taurine supplementation, L-carnitine was administered concurrently and with clinical success [7]. Therefore, whether or not taurine supplementation alone would be sufficient to result in the clinical improvement observed in this study could not be determined. However, several dogs in this study showed clinical and echocardiographic improvement without L-carnitine supplementation, suggesting that this may not be required in all cases. No differences were noted between dogs that were treated with taurine supplementation alone and dogs treated with both L-carnitine and taurine supplementation. Further controlled prospective studies would need to be performed to determine the relevance of L-carnitine in treatment of taurine deficient DCM.

In addition, sample collection and the methodology used for taurine analysis could not be standardized as this was an observational study mimicking clinical practice. For example, taurine analysis was performed at multiple reference laboratories and either plasma, whole blood, serum, or a combination of whole blood and plasma taurine concentrations were obtained. Currently, it is recommended that both whole blood and plasma taurine concentrations be obtained together, and that a diagnosis of taurine deficiency be made if either of those values are low. Serum has been shown in cats to be less repeatable and therefore, is not considered the gold standard for measuring taurine concentrations [48]. In addition, improper sample handing can result in falsely high (plasma) or low (whole blood) concentrations of taurine. Further, it is not known how accurately canine whole blood or plasma taurine concentrations reflect taurine concentrations in cardiac tissue. A 2001 study by Pacioretty et al determined that in sixteen taurine depleted cats, whole blood taurine concentrations appeared to more accurately reflect taurine concentrations in the skeletal muscle during taurine depletion, while plasma taurine concentrations more accurately reflected skeletal muscle taurine concentrations during taurine repletion [48]. Further studies are required to determine if taurine concentrations behave similarly in dogs. Because obtaining myocardial tissue for taurine measurement is not clinically practical, skeletal muscle biopsies may be considered as an acceptably accurate method of assessing whole body taurine status. Further studies are required.

Currently, reference ranges for normal taurine concentrations as used in this study are not breed or size-specific. It is important to note that all-breed reference values established for taurine concentrations may not be appropriate for all dogs. This highlights the importance of establishing reference values in golden retrievers specifically to more accurately identify which golden retrievers are susceptible to developing nutritionally-mediated DCM. The fact that some of the golden retrievers in our study with DCM had whole blood taurine concentrations between 200–250 nmol/mL suggests that reference ranges may vary depending on the breed or individual dog, and for the golden retriever may be higher than expected. The reference ranges utilized in this study are supported by the literature but suggest that commercial laboratory values may require reevaluation [13, 14]. It is our current recommendation that in golden retrievers, taurine deficiency be considered at whole blood and plasma taurine concentrations of ≤ 250 nmol/ml and ≤ 60 nmol/ml, respectively. Additional studies are required to confirm the most appropriate reference ranges in the golden retriever to determine which dogs are at greatest risk for developing DCM. Importantly this study did not assess the true prevalence of taurine deficiency in the client-owned golden retriever population and as such the reported prevalence in this manuscript may represent an over or under representation of the population. This represents a future aim for prospective studies.

## Conclusions

All dogs with DCM in this study were consuming diets with similar characteristics, including grain-free, uncommon protein based, or legume-rich formulations. We also noted that all but one of these dogs were consuming less food than their calculated MER would suggest and thus cannot exclude the role of dietary intake in these cases. In addition, we identified an increased prevalence of taurine deficiency in a sample of apparently healthy golden retrievers fed similar diets. Although a cause and effect relationship cannot be proven, the associations are concerning and warrant caution as well as future prospective studies. Assessment of taurine status of golden retrievers consuming similar diets may be indicated; however, adequate blood concentrations of taurine may be higher than previously recognized for some dogs. This data also underscores the immense value of obtaining complete diet histories in canine patients with cardiac disease.

Future, controlled, prospective studies of larger sample size are needed to determine if the clear associations identified in this manuscript represent a cause-and-effect relationship between DCM, taurine-deficiency, specific ingredients, and grain-free food varieties in general. Without such studies we cannot conclusively define which dietary characteristics are involved with the pathogenesis of this condition.
