# Correction: Taurine deficiency and dilated cardiomyopathy in golden retrievers fed commercial diets

**DOI:** 10.1371/journal.pone.0210233

**Published:** 2018-12-31

**Authors:** Joanna L. Kaplan, Joshua A. Stern, Andrea J. Fascetti, Jennifer A. Larsen, Hannah Skolnik, Gordon D. Peddle, Richard D. Kienle, Andrew Waxman, Michael Cocchiaro, Catherine T. Gunther-Harrington, Tyler Klose, Kendra LaFauci, Bonnie Lefbom, Maggie Machen Lamy, Rebecca Malakoff, Satoko Nishimura, Maureen Oldach, Steven Rosenthal, Christopher Stauthammer, Lynne O’Sullivan, Lance C. Visser, Regan Williams, Eric Ontiveros

The twenty-second author's name is spelled incorrectly. The correct name is: Regan Williams.

There is also an error in Table 2. Row 8L, columns G and L should not have check marks in them. Please see the corrected Table 2 here.

**Table 2:** List of pet food brands with their diet varieties and characteristics. For each pet food variety, the number of dogs diagnosed with DCM fed this diet and the number of dogs with taurine deficiency fed this diet were listed. G = grain-free diet, L = if a legume is listed as one of the first five ingredients of the diet. Note that one dog on diet 1a is the same dog receiving diet 9m. The one dog receiving diet 8k is the same dog receiving diet 8l.

Diet Brand	Diet Variety	No. of dogs with DCM	No. of dogs with low Taurine	Feeding Trial Tested	Formulated to Meet AAFCO Guidelines	Meets WSAVA Guidelines	G	L
1	a b c	10 4 1	10 4 1	No No No	✓ ✓ ✓	No No No	✓ ✓ ✓	✓ ✓
2	d	1	1	No	✓	No	✓	✓
3	e f	1 1	1 1	No No	✓ ✓	No No	✓ ✓	✓ ✓
4	g	1	1	No	✓	No	✓	✓
5	h	2	2	No	✓	No	✓	✓
6	i	1	1	No	✓	No	✓	✓
7	j	1	1	No	✓	No	✓	✓
8	k l	1 1	1 1	No No	✓ ✓	No No	✓	✓
9	m	1	1	No	✓	No	✓	

1a = ACANA Singles Limited Ingredient Diet Pork & Squash Formula (dry); Manufactured by Champion Petfoods USA Inc., Auburn, KY 42206.

1b = ACANA Singles Limited Ingredient Diet Lamb & Apple Formula (dry); Manufactured by Champion Petfoods USA Inc., Auburn, KY 42206.

1c = ACANA, unknown variety; Manufactured by Champion Petfoods USA Inc., Auburn, KY 42206.

2d = Taste of the Wild Pet Food Pine Forest Canine Recipe with Venison & Legumes (dry); Schell & Kampeter, Inc. Manufactured by Diamond Pet Foods.

3e = 4health Grain Free Beef & Potato Formula (dry); Tractor Supply Co.; Manufactured by Ainsworth Pet Nutrition, Meadville PA 16335.

3f = 4health Grain Free Chicken & Vegetable Formula (dry); Tractor Supply Co.; Manufactured by Diamond Pet Foods.

4g = Zignature Lamb Formula Limited Ingredient (dry); Pets Global, Inc.; Manufactured by Tuffy’s Pet Foods, Perham, MN 56573.

5h = Instinct Limited Ingredient Diet Grain-Free Recipe with Real Lamb (dry); Nature’s Variety, Saint Louis, MO 63146. Manufactured by CJ Foods, Inc, Bern, KS, 66408.

6i = NutriSource Grain Free Chicken and Peas Formula (dry); Manufactured by KLM Family Brands, Tuffy’s Pet Foods, Perham, MN 56573.

7j = Kirkland Signature Nature’s Domain, Turkey Meal & Sweet Potato Formula for Dogs (dry); Manufactured by Diamond Pet Foods.

8k = Fromm Lamb & Lentil Recipe Dog Food (dry); Manufactured by Fromm Family Foods LLC, Mequon WI 53092

8l = Fromm Salmon a La Veg (dry); Manufactured by Fromm Family Foods LLC, Mequon WI 53092

9m = Orijen Regional Red (freeze-dried medallions); Manufactured by Champion Petfoods USA Inc., Auburn, KY 42206.

(✓) Diet meets criteria under the specific category
